# The Combinatory Effect of Spirulina Supplementation and Resistance Exercise on Plasma Contents of Adipolin, Apelin, Ghrelin, and Glucose in Overweight and Obese Men

**DOI:** 10.1155/2022/9539286

**Published:** 2022-06-13

**Authors:** Malekaneh Mohammad, Dehghani Karim, Mogharnasi Mehdi, Saghebjoo Marziyeh, Sarir Hadi, Nayebifar Shila

**Affiliations:** ^1^Department of Clinical Biochemistry, School of Medicine, Birjand University of Medical Sciences, Birjand, Iran; ^2^Department of Exercise Physiology, Faculty of Sport Sciences, University of Birjand, Birjand, Iran; ^3^Department of Animal Sciences, University of Birjand, Birjand, Iran; ^4^Department of Sport Sciences, Faculty of Educational Sciences and Psychology, University of Sistan and Baluchestan, Zahedan, Iran

## Abstract

**Methods:**

The current investigation was conducted in a single-blind and quasiexperimental fashion. Sixty overweight and obese men (BMI > 25) ranging in age from 30 to 55 years were purposefully selected and randomly assigned to one of four groups: training plus spirulina (T+S), training plus placebo (T+P), spirulina (S), or placebo (P). For eight weeks, the (S) and (P) groups consumed two 500 mg spirulina and placebo capsules daily, respectively. Resistance training was performed three sessions a week over eight weeks, consisting of 12 movements with 1-, 2-, 3-, and 4-minute rest intervals and 40-90 percent maximal repetition. Adipolin, apelin, and ghrelin indices were measured before and after exercise using special kits.

**Results:**

All variables changed significantly between groups except for apelin. Within-group comparisons revealed a substantial increase in adipolin levels in the (T+S) and (T+P) groups (*P* < 0.05). Apelin levels were decreased in the (T+S) and (T+P) groups. Additionally, FBS levels reduced significantly in (T+S) (*P* = 0.01).

**Conclusion:**

It seems that eight weeks of circuit resistance training and spirulina supplementation can lead to reduced weight and apelin and FBS levels as well as increased concentrations of adipolin and ghrelin contents in overweight and obese men.

## 1. Introduction

Overweight and obesity are major global issues escalating in many societies [1–4]. Exercise is well recognized as a strategy that not only lessens inflammatory factors but also benefits weight loss by significantly reducing energy, nutrient balance, and body function [5]. Adipose tissue (AT) secretes a biologically active substance called adipokine, which regulates both energy metabolism and the intricate interactions between AT and bone [6, 7]. One of these vital regulators is adipolin, an anti-inflammatory cytokine that is predominantly generated and secreted in AT and can be decreased in obesity and other pathological conditions associated with obesity [8, 9]. Adipolin is found in white adipose tissue, and its expression is moderately regulated in obese individuals [10, 11]. However, additional factors have been found to affect the function and energy of human glucose homeostasis, which appears to be an AT-released inflammatory indicator [6, 7, 12]. When apelin binds to its receptor, it is phosphorylated, resulting in NO release via L-arginine and an increase in the amount of cyclic guanosine monophosphate (cGMP) [13]. However, in the presence of the abnormal and malfunctioning endothelium, apelin acts directly on vascular smooth muscle cells, activating the APJ receptor and causing vasoconstriction [14].

Bodyweight regulation is a highly complex and precisely controlled process, where hormones are secreted by AT and the stomach, affecting the functioning of energy homeostasis centers and causing weight change by changing appetite [15].

As a peptide hormone secreted in large amounts by the stomach, ghrelin increases under negative energy balance conditions such as hunger and weight loss and decreases at positive energy levels such as obesity and food intake [16]. However, calorie restriction and diet are the primary therapeutic interventions in controlling weight gain. Meanwhile, physical activity and exercise are also recommended as helpful behavioral interventions in modulating inflammatory mediators. Substantial research has explored the influence of exercise on cellular fat levels to contribute to treating obesity-related disorders by elucidating the mechanism through which these fats are modulated [17, 18].

Given the high prevalence of obesity and its detrimental consequences on human health, nutritional diets and physical activities have been recently postulated as effective weight management interventions. Resistance training is an effective strategy in losing weight and improving obesity-related disorders [19]. Resistance training improves muscle tone and translation activity, boosts structural and contractile protein production, and stimulates satellite cell proliferation, myoblast secretion, and physiological factors [20]. Like other eastern nations, Iranians are willing to employ diets or herbal therapies to expedite the weight loss process. In these circumstances, it makes sense to find the appropriate supplementary treatment.

Lately, herbal supplements have been used as a strategy in weight management. Spirulina is a species of microalgae that contains minerals, vitamins such as B12, protein A (*β*-carotene), phenolic acids, *γ*-linoleic acid, and tocopherol. The herb is found to lower fatty liver, chances of cardiovascular disease, and serum lipid levels in diabetic and obese patients [21]. Regular intake of spirulina has been shown to influence proinflammatory cytokine expression and NF-*κ*B translocation, hence lowering the risk of inflammatory and obesity-related disorders via increased macrophage infiltration [22, 23]. To the best of the authors' knowledge, no studies have examined the effectiveness of circuit resistance training and spirulina as measures to treat inflammatory factors in overweight and obese men. Therefore, this study is aimed at investigating the effect of eight weeks of circuit resistance training and spirulina supplementation on plasma levels of adipolin, apelin, ghrelin, and glucose in overweight and obese men.

## 2. Materials and Methods

### 2.1. Study Population

The current study used a single-blind, quasiexperimental design with a pretest and a posttest. A sample size of sixty overweight (25 > BMI ≥ 29.99 kg/m^2^) and obese males (30 ≥ BMI ≥ 34.99 kg/m^2^) was selected. The inclusion criteria comprised the absence of any disease, including cardiovascular disease, hormone abnormalities, kidney, or liver disease, as well as a history of surgery, herbal or therapeutic diets, diabetes, cancer, smoking, hyperlipidemia, and hypertension (obesity or surgery drugs). The exclusion criteria were nonparticipation in training, vitamin and mineral supplementation, and injury sustained while training. On the laboratory visit, required information such as age and height was collected. Individuals were informed thoroughly of the research procedures and potential risks. They completed the written Physical Activity Readiness Questionnaire (PAR-Q) and signed a consent form voluntarily. After that, the participants were assigned to four equal groups of fifteen individuals based on a simple random allocation method (alternative): T+S (*n* = 15), T+P (*n* = 15), S (*n* = 15), and P (*n* = 15). The local ethics committee ethically approved the current investigation, and the study protocol followed the Helsinki Declaration (IR.BUMS.REC.1398.046).

### 2.2. Spirulina Supplementation Procedure

Spirulina supplement was supplied from Reyhan Naghsh Jahan Pharmaceutical Company with product registration license (IRC908021898759013) and license number 9080218987590713 issued by the Iranian Health Ministry's Deputy of Food and Drug. Similarly, the placebo capsule was provided from Nader Isfahan Company with registration number 14906. Spirulina supplements were supplied in packs of 60 dark green capsules weighing 500 mg. For two months, the (S) and (T+S) groups received spirulina pills (500 mg) before and after lunch (weight dose recommended by the manufacturer), along with 150 ml of water twice daily (9 a.m. and 3 p.m.). Additionally, the (T+P) and (P) groups received two 500 mg capsules (containing starch) with 150 ml of water that resembled the spirulina capsules in appearance, weight, and packaging [24]. Weekly telephone interviews were used to verify adherence to the protocols and monitor any complications.

### 2.3. Nutritional Supplement Intake on Diet

Research findings indicate that diet control is associated with specific outcomes [25]. Hence, the subjects' diets were monitored using a food frequency questionnaire (FFQ) throughout the investigation. Individuals' diets were examined using a validated Iranian-specific FFQ. The researchers recorded nutritional information in the last exercise session each week using questionnaires. The study drew on the USDA Food Pyramid to determine the food codes. The average energy consumption of macronutrients (carbohydrates, proteins, and fats) was computed weekly using the Nutritionist4 software (Versions 2, 5, and 3), which was programmed using Delphi 7 programming.

### 2.4. The Exercise Protocol

The resistance training program was conducted using Fleck's model [26]. The subjects exercised three 80- to 90-minute sessions per week held at 5 to 6:30 p.m. for eight consecutive weeks. Each training session shared the same environmental conditions (~20°-24°C and ~55% humidity). Accordingly, resistance training consisted of 12 movements, whose details are provided in Tables 1 and 2 [27]. Participants performed 10 minutes of warm-up and cool-down at the beginning and end of each exercise session. The one-repetition maximum was determined by the following equation [28]. It should be noted that the same protocol and program design have been performed on the same participants in the researchers' previous Persian papers [29–31].

(1) $$\begin{array}{c} \text{One}‐\text{repetition maximum}=\frac{\text{Weight lifted}\left(\text{kg}\right)}{1.0278‐\left(\text{Repeats up to exhausting}\times 0.0278\right)}. \end{array}$$

### 2.5. Biochemical Analysis

Ten milliliters of blood was collected from the left arm vein after 12 hours of fasting in the morning at the start and end of the eight-week intervention (48 hours). The blood sample was then centrifuged at 3500 rpm for 10 minutes using a device (ROTOFIX32A Hettich, Germany). The plasma was separated at room temperature, and the samples were immediately refrigerated at -80°C. Plasma adipolin, ghrelin, and apelin levels were measured using a sandwich ELISA kit (Zell Bio Co, Germany; sensitivity = 0.05 ng/m) and by the ELISA Reader (US Liosion Model). Due to a shortage of kit wells, 11 individuals were assayed for adipolin, apelin, and ghrelin indices. A trained laboratory assistant performed all biochemical assays, and all measures were taken in the hormone laboratory of Imam Reza hospital in Birjand city.

### 2.6. Statistics

Baseline parameters did not differ significantly between groups. The Shapiro-Wilk test assessed the normal distribution of the data. Moreover, the data were compared using a paired *t*-test before and after the interventions. Differences between groups were assessed using a one-way analysis of covariance. A post hoc LSD interactive analysis was employed to identify discrepancies. The level of significance was set at *P* < 0.05. All data were analyzed using the Statistical Package for the Social Sciences (SPSS Inc., Chicago, IL, USA) software version 24.0 and were expressed as means ± standard deviation.

## 3. Results

**Figure 1:**
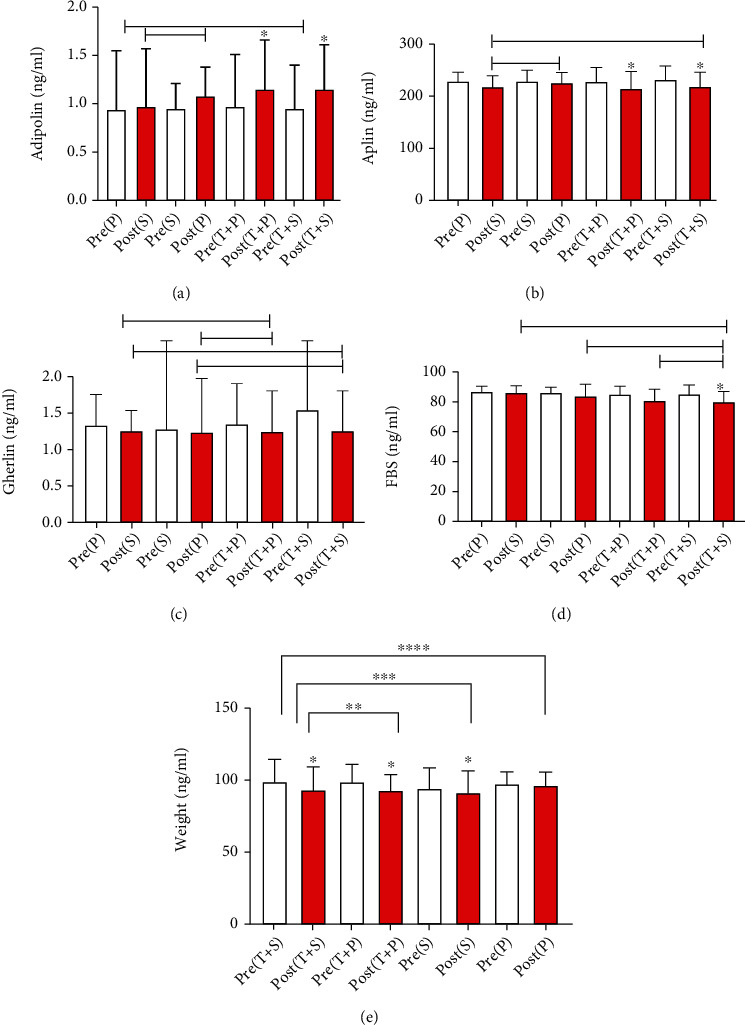
Between- and within-group changes of (a) adipolin, (b) aplin, (c) gherlin, (d) FBS, and (e) Weight.

There was no significant difference between the groups in terms of carbohydrate (*P* = 0.77), protein (*P* = 0.58), and fat (*P* = 0.88) intake (Table 3). Demographic characteristics of participants in groups are shown in Table 4. Tables 5 and 6 show there was a significant difference in the levels of adipolin (*P* = 0.04), ghrelin (*P* = 0.01), weight, and FBS (*P* = 0.03) between groups, while no between-group differences were found in apelin (*P* = 0.31). The results revealed a significant difference in adipolin between (T+S) and (P) groups (*P* = 0.04) and between the (S) and (P) groups (*P* = 0.01). Moreover, ghrelin levels differed between (T+S) and (S) (*P* = 0.01), between (T+S) and (P) (*P* = 0.01), between (T+P) and (S) (*P* = 0.01), and between (T+P) and (P) (*P* = 0.01) groups. Weight differed between (T+S) and (S) (*P* = 0.01), between (T+P) and (P) (*P* = 0.01), and between (S) and (P) (*P* = 0.37). FBS had a significant difference between (T+P) and (S) (*P* = 0.02) and between the (S) and (P) groups (*P* = 0.01). Within-group differences showed an increase in adipolin levels in (T+S) (*P* = 0.01) and (T+P) (*P* = 0.05) groups (Figure 1(a)). However, apelin levels decreased in the (T+S) (*P* = 0.01) and (T+P) (*P* = 0.01) groups (Figure 1(b)). Lastly, weight improved in the (T+S) (*P* = 0.01), (T+P) (*P* = 0.01), and placebo groups (*P* = 0.03) (Figure 1(e)), while FBS declined in (T+S) (*P* = 0.01) (Figure 1(d)). Nevertheless, ghrelin levels did not change significantly within groups (*P* > 0.05) (Figure 1(c)).

## 4. Discussion

This study intended to evaluate the effect of eight weeks of circuit resistance training and spirulina supplementation on plasma levels of adipolin, apelin, ghrelin, and glucose in overweight and obese men. According to Tables 5 and 6, there was a significant difference in adipolin levels between the (T+S) and (P) groups and between the (S) and (P) groups, while apelin concentrations were not significantly different across groups. Moreover, ghrelin levels differed between (T+S) and (S), between (T+S) and (P), between (T+P) and (S), and between the (T+P) and (P) groups. FBS was significantly different between (T+P) and (S) and between the (S) and (P) groups. Within-group changes showed an increase in adipolin concentrations in the (T+S) and (T+P) groups. Apelin levels decreased in the (T+S) and (T+P) groups and FBS in (T+S). However, ghrelin levels did not change significantly across groups (*P* > 0.05) (Table 5).

Our findings concerning adipolin levels are consistent with those of Rahmatollahi et al. and Rezaian et al. (2016) but inconsistent with those of Suri et al. and Rezaian et al. [32–35]. These discrepancies may be affected by the duration and intensity of the training periods, the training protocols, and the subjects' race and gender, among others. After ten weeks of aerobic exercise, Suri et al. found no significant change in adipolin plasma levels and insulin resistance in overweight men. Likewise, Rezaian et al. assessed the long-term effectiveness of 12-week acute endurance and resistance training on serum adipolin levels in sedentary postmenopausal women, findings that the training was effective though nonsignificantly. The present study results are inconsistent with the findings reported by Rezaian et al. and Syrian et al. The protocols and subjects may explain the difference between the results of these two studies and the current research.

Obesity-linked stresses negatively affect adipolin expression, decreasing adipolin levels after exercise [36]. A systematic review demonstrated that prolonged endurance, resistance, and high-intensity interval training can reduce the circulating contents of proinflammatory cytokines (IL-6, CRP, and TNF-*α*). In contrast, resistance training primarily enhances anti-inflammatory cytokines (IL-10). This regulation in low-grade systemic inflammation seems independent of exercise-induced fat mass loss [37]. A set of mechanisms are involved in an exercise leading to modified insulin resistance, reduced fats, and increased oxidation. They are as follows: increasing insulin receptor, glucose, and mRNA transporter protein (GLUT4); enhancing glycogen synthetase and protein kinase-B and hexokinase; improving the function of intracellular insulin messages; mediating molecules on insulin signals including increased ERK2 expression, PI3K activity, and AMPK signal; effecting changes in muscle composition (increasing capillary density in muscle fibers and converting muscle fibers to rapid oxidative contractile fibers); increasing glucose delivery to muscles; and reducing triglyceride accumulation in muscle cells and acid secretion. Length and intensity are two characteristics of exercise that strongly affect the insulin response to exercise. It seems that despite the inverse relationship between glucose and adipolin, significant alterations in glucose can be one of the factors affecting adipolin changes [32].

In this study, plasma apelin levels decreased significantly after eight weeks of resistance training. Increased adrenal secretion by adipose tissue can be effective in various obesity-related disorders [38]. According to Eldor and Raz, apelin is released from fat in response to food or insulin stimulation [39]. Some studies have indicated a positive relationship between apelin circulation and BMI [6]. After 12 weeks of diet-induced weight loss, apelin levels in obese women are reported to decline [6]. Additionally, weight loss inhibits apelin gene expression [40]. Inconsistent with the present study, Zhang et al. showed a rise in apelin concentration in overweight individuals after 12 weeks of aerobic exercise [41]. The discrepancy between these results and those of the current study can be attributed to a variety of factors, including differences in research groups, length of training, the intensity of training, and type of training. Sheibani et al. demonstrated that endurance exercise lowers apelin levels in obese women [42], which reinforces the findings of our investigation.

In the present study, the bodyweight of the exercising groups was significantly reduced. It has been shown that during physical activity and exercise, the body's endocrine system increases fat oxidation (lipolysis) by boosting epinephrine, norepinephrine, growth hormones, and cortisol. Moreover, it releases free fatty acids to produce energy for muscles during exercise, thereby the body losing its fat mass [43].

Various studies have revealed a significant inverse relationship between regular physical activity and inflammatory markers. It has been found that those who are more physically active and in better physical shape have lower inflammatory marker levels, which is associated with their anti-inflammatory properties [44, 45]. Therefore, if exercise fails to reduce adipose tissue or improve the function of these cells, the effect of exercise on adipokine level modulation, insulin resistance, and inflammation cannot be expected [46]. Resistance training is linked with metabolic and endocrine changes and helps to develop the function of obese people by reducing body fat mass. Increased blood flow to adipose tissue during resistance exercise also helps alleviate tissue hypoxia and inflammatory diseases [47]. The current study's findings indicate that apelin levels in the plasma are dramatically decreased in addition to improved body composition indicators. The significant reduction in apelin was most likely owing to improved physical parameters and a further decrease in fat percentage, which may be linked with body fat loss as a mechanism of apelin production. Foldes et al. consider that the source of apelin gene expression is a part of the apelin cardiovascular system [12], whereas Castan et al. argue that increasing adipose tissue might potentially provide apelin to the circulatory cycle [7].

Although ghrelin is known as an endogenous factor for growth hormone-secreting receptors, it is involved in regulating food consumption behavior, energy homeostasis, and weight regulation through growth hormone-independent mechanisms [48]. Findings from studies on the effect of long-term exercise on ghrelin levels have also shown that activities that lead to weight loss significantly increase total plasma ghrelin levels [49]. Given the impact of ghrelin in controlling weight and appetite, this peptide seems to be affected by exercise. Research on the effects of physical activity on changes in plasma ghrelin levels has reported contradictory results. In some studies, the plasma ghrelin level has increased [50, 51]; in others, it has decreased [48]. Some researchers have also noted that ghrelin levels do not change [52–54]. Studies have shown that acute resistance reduces appetite by temporarily reducing ghrelin concentration [55]. The findings of this study contradict the findings of Jafari et al., which showed that eight weeks of resistance training did not affect appetite-related hormones such as ghrelin [56].

The role and importance of spirulina supplementation in these changes should not be overlooked. Studies have shown that spirulina supplementation is involved in lipid and blood glucose metabolism [57]. The easy absorption of sugar in spirulina stabilizes blood sugar and prevents the craving for certain foods in people. Hence, having a diet with spirulina herbal supplements reduces body weight balance and the need for insulin. Overall, spirulina intake not only improves the abnormal sugar levels but may also increase the body's ability to reduce inflammation in obese people [57]. It seems that the antioxidant role of spirulina is to restore equilibrium to obesity-induced oxidative stress. It is also an influential factor in lipid metabolism and casts a more substantial impact when coupled with resistance training than the impact exerted by exercise alone. Therefore, according to the findings of this study, it seems that spirulina supplementation is effective on body composition and physical fitness factors of overweight and obese people, contributing to a reduction in visceral fat and the prevention of liver fat accumulation.

A previous study has demonstrated that supplementation and its association with strength training promote intestinal contractile reactivity and oxidative stress [58]. Another research admitted the alleviating effects of spirulina supplementation and strength training for eight weeks (in a dose-effect manner) on inflammation (CRP) [59]. By modulating HDACs and histone acetylation and inhibiting the NF-*κ*B pathway, spirulina reduces the production of proinflammatory cytokines such as TNF-*α*, IL-1*β*, and IL-6 [60]. Indeed, it ameliorates visceral adipose tissue macrophages by decreasing percent body fat [61]. However, TNF-*α*, IL-1*β*, IL-6, and NF-*κ*B were not measured in the present research.

The effect of resistance training on body composition and increased muscle mass is well known and one of the ways to reduce overweight and obesity [62]. Further research is required to confirm the results of this research. Besides, since the subjects were human, the role of diet control in achieving different outcomes should not be overlooked. However, these differences may be rooted in the training duration and intensity, the training protocol, and the participants' race/gender. The results of this study faced limitations, such as the length of the training period. The researchers tried to control the participants' diet and avoid any regular exercise other than the study exercise program. Nonetheless, it is hardly possible to accurately control these cases in humans.

## Figures and Tables

**Table 1:** Weekly program of resistance training protocol.

Workout sequence	1	2	3	4	5	6	7	8
Day 1	L	L	M	VL	M	L	VL	H
Day 2	M	VL	H	H	M	M	M	VL
Day 3	L	H	L	L	L	H	L	M

L: light-intensity workout; M: moderate-intensity workout; VL: very light-intensity workout; H: heavy-intensity workout. There was an active rest day after any workout.

**Table 2:** Nonlinear resistance training protocol.

Exercises	Very light	Style	Average	Heavy
Knee extension	20/40 × 1	15/60 × 2	10/75 × 3	4/90 × 4
Bench press	20/40 × 1	15/60 × 2	10/75 × 3	4/90 × 4
Incline bench press	20/40 × 1	15/60 × 2	10/75 × 3	4/90 × 4
Seated row	20/40 × 1	15/60 × 2	10/75 × 3	4/90 × 4
Deadlift	20/40 × 1	15/60 × 2	10/75 × 3	4/90 × 4
Pulley crunches	20/40 × 1	15/60 × 2	10/75 × 3	4/90 × 4
Lat pull-downs	20/40 × 1	15/60 × 2	10/75 × 3	4/90 × 4
Calf raise	20/40 × 1	15/60 × 2	10/75 × 3	4/90 × 4
Hamstring curl	20/40 × 1	15/60 × 2	10/75 × 3	4/90 × 4
Press behind neck	20/40 × 1	15/60 × 2	10/75 × 3	4/90 × 4
Upright row	20/40 × 1	15/60 × 2	10/75 × 3	4/90 × 4
Arm curl	20/40 × 1	15/60 × 2	10/75 × 3	4/90 × 4

Length of rest period: very light: 1 minute; light and moderate: 1-2 minutes; heavy: 3-4 minutes; and very heavy: 5-7 minutes. 1 set, 320 repetitions, 40% 1RM.

**Table 3:** Comparison of macronutrients (carbohydrates, proteins, and fats) contents in groups.

Variable	T+S (mean ± SD)	T+P (mean ± SD)	S (mean ± SD)	P (mean ± SD)	*P* ANOVA
Carbohydrate	55.780 ± 1.920	56.446 ± 0.985	56.612 ± 2.128	56.264 ± 2.450	0.776
Protein	17.488 ± 0.608	17.795 ± 0.775	17.651 ± 0.665	17.853 ± 1.008	0.582
Fat	26.732 ± 0.988	25.759 ± 1.009	25.759 ± 2.859	25.883 ± 2.859	0.883

**Table 4:** Demographic characteristics of participants in groups.

Variable	T+S (mean ± SD)	T+P (mean ± SD)	S (mean ± SD)	P (mean ± SD)
Age (years)	36.000 ± 6.199	37.066 ± 6.441	39.33 ± 10.965	35.400 ± 8.716
Height (m)	1.728 ± 0.055	1.730 ± 0.096	1.718 ± 0.082	1.732 ± 0.064

**Table 5:** Comparison of within- and between-group changes of variables.

Variable (^∗^)	Group (mean ± SD)	Pretest	Posttest	*F*	df	Eta squared	*P* ANOVA	Observed power	*P* (paired *t*)
Adipolin (ng/ml)	T+S	0.95 ± 0.46	1.15 ± 0.47	1.00	3	0.60	^∗^0.04	1.00	^∗^0.01
	T+P	0.97 ± 0.55	1.10 ± 0.58						^∗^0.01
	S	0.95 ± 0.27	1.08 ± 0.31						0.21
	P	0.94 ± 0.62	0.97 ± 0.61						0.34

Apelin (ng/ml)	T+S	229.69 ± 27.57	216.60 ± 28.95	220.21	3	0.33	0.31	0.40	^∗^0.01
	T+P	226.11 ± 27.78	212.62 ± 34.28						^∗^0.01
	S	226.52 ± 22.99	223.28 ± 21.77						0.21
	P	217.57 ± 27.75	207.91 ± 30.44						0.12

Ghrelin (ng/ml)	T+S	1.23 ± 0.58	1.44 ± 0.61	1.33	3	0.72	^∗^0.01	0.72	0.40
	T+P	1.22 ± 0.9	1.32 ± 0.59						0.75
	S	1.21 ± 0.77	1.25 ± 0.79						0.67
	P	1.23 ± 0.31	1.30 ± 0.46						0.47

FBS (mg/dl)	T+S	84.26 ± 7.06	79.20 ± 7.66	81.65	3	0.69	^∗^0.03	0.81	^∗^0.01
	T+P	84.13 ± 6.33	80.06 ± 8.28						0.11
	S	85.20 ± 4.49	83.06 ± 8.75						0.28
	P	85.86 ± 4.51	85.33 ± 5.44						0.73

**Table 6:** Bonferroni's multiple comparison test for variables.

Variable (^∗^)	Multiple comparisons (LSD)	
	Groups	*P*
Adipolin (ng/ml)	T+S vs. P S vs. P	^∗^0.04 ^∗^0.01
Ghrelin (ng/ml)	T+S vs. S T+S vs. P T+P vs. S T+P vs. P	^∗^0.01 ^∗^0.01 ^∗^0.01 ^∗^0.01
FBS (mg/dl)	T+P vs. S S vs. P	^∗^0.02 ^∗^0.01

## Data Availability

All data may be made available from the corresponding author upon reasonable request.
